# Blind free-living kiwi offer a unique window into the ecology and evolution of vertebrate vision

**DOI:** 10.1186/s12915-017-0424-0

**Published:** 2017-09-15

**Authors:** Bret A. Moore, Joanne R. Paul-Murphy, Alan J. D. Tennyson, Christopher J. Murphy

**Affiliations:** 1 William R. Pritchard Veterinary Medical Teaching Hospital, School of Veterinary Medicine, University of California-Davis, 1 Garrod Drive, Davis, CA 95695 USA; 2 Department of Medicine and Epidemiology, School of Veterinary Medicine, University of California-Davis, One Shields Avenue, Davis, CA 95616 USA; 3 Museum of New Zealand Te Papa Tongarewa, P.O. Box 467, Wellington, New Zealand; 4 From the Department of Surgical and Radiological Sciences, School of Veterinary Medicine, University of California-Davis, One Shields Avenue, Davis, CA 95616 USA; 5 Department of Ophthalmology & Vision Science, School of Medicine, University of California, 2315 Stockton Blvd, Sacramento, CA USA

## Abstract

The first report of multiple, blind, wild birds in good health suggests vision is not necessary for the survival of kiwi.

The avian visual system is widely recognized for its superior performance [1]. Birds are broadly considered to have an unmatched dependence for high quality visual information compared to other vertebrate classes, largely due to a unique set of ecological demands (e.g., flight, foraging behavior, and sexual behavior) [1, 2]. The typical avian eye is relatively conserved in that regard, stamped with several distinctions that afford high performance, such as large size and densely packed retinal neurons [2, 3]. However, dramatic interspecific variation is also present, enabling different species to perform visually at a high level while occupying ecological niches with different challenges, such as variable habitat types and activity patterns [2].

Although strict nocturnality in birds is uncommon, most avian species active in low light levels have specializations within their visual systems that facilitate nocturnal performance (e.g., large pupillary aperture for gathering light, rod-dominated retinae for high light sensitivity). However, kiwi (*Apteryx* spp.), a nocturnal and flightless group of birds, do not follow this general pattern. They possess the smallest eyes relative to body mass of any avian species, have underrepresented visual brain regions, and have the smallest visual fields among birds [4, 5]. It is likely that the visual system of kiwi is only able to coarsely resolve objects within its visual field in a nocturnal environment [5]. This surprisingly mundane set of visual characteristics for a bird becomes understandable when one considers the kiwi’s energetic devotion and input to other sensory systems: auditory [6], olfactory [7], and tactile somatosensory systems with a uniquely positioned set of mechanoreceptors at the tip of their long bill [4, 7].

With such prowess among other sensory systems, one may ask whether vision in kiwi is necessary for survival? We offer evidence that it indeed is not, and describe the first report of complete blindness or severe visual compromise affecting more than one individual in a free-living avian population where physical condition was not significantly impacted. Through an intensive conservation management program, an ophthalmologic survey was performed on 160 free-living Okarito kiwi (*Apteryx rowi*) in their natural habitat in New Zealand. Initial brief examinations revealed that about one-third of the birds had ocular lesions in one or both eyes (Table 1). A detailed examination was performed by a veterinary ophthalmologist (CJM) on 11 of the kiwi with these lesions. Common abnormal findings included corneal opacification (e.g., edema, fibrosis), shrunken fibrotic globes (phthisis bulbi), among others (Fig. 1). Such ophthalmic findings are prevalent in captive avian populations, but are rare in free-living prey species with reports being limited mostly to raptors (i.e., eagles, hawks, owls, and other predatory birds) [2]. This is likely due to the profound negative impact on survival that any reduction in the quality of visual information can have on visually dependent species, which is shown here to not be the case for kiwi, who may depend on other sensory systems.

**Table 1:** Distribution of ocular lesions recorded in 53/160 monitored kiwi

Lesion description	Unilateral	Bilateral	Total eyes
Buphthalmia	0	1	1
Phthisis bulbi	3	0	3
Corneal opacity	4	16	20
Iridal abnormality	5	1	6
Other ocular lesion	3	20	23
Complete blindness	1	4	5

**Fig. 1.**
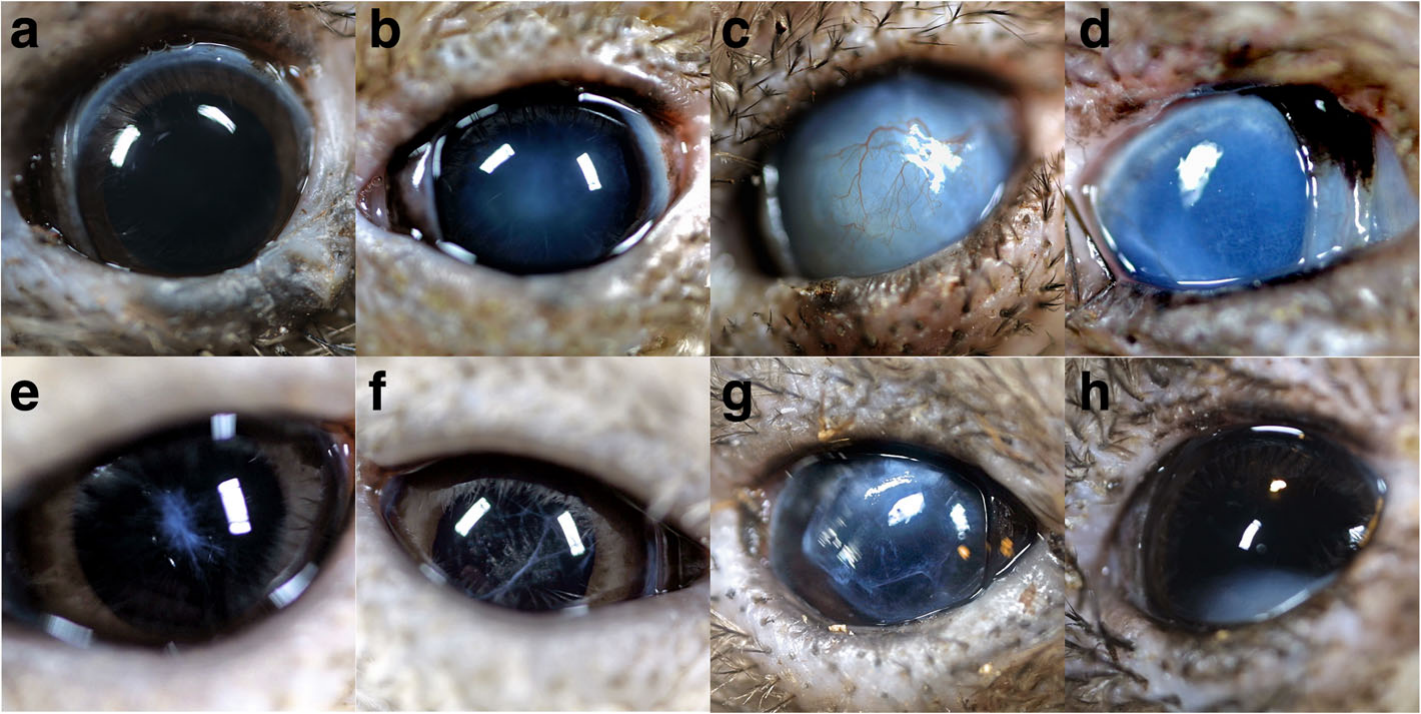
Normal and pathologic findings for the anterior segment of the Okarito brown kiwi. Complete ophthalmic examinations consisted of slit lamp biomicroscopy, direct ophthalmoscopy, and streak retinoscopy. Lack of vision was interpreted by no response to light or motion, combined with the severity of ocular lesions (e.g. inability to visualize intraocular structures beyond the abnormal ocular tissue, such as marked corneal or lens opacification). **a** Normal anterior segment. Note the small palpebral aperture (mean diameter 8.53 ± 0.50 mm SD, n = 9 birds). **b** Nuclear sclerosis: a normal aging change in the lens associated with changes in lens protein composition. Nuclear sclerosis generally has minimal visual consequences in animals. **c** Buphthalmia with marked corneal edema. This animal was blind bilaterally but was in good physical condition. **d** Phthisis bulbi (a globe shrunken with fibrosis). Potential causes include any chronic inflammatory or glaucomatous process or severe trauma. **e**, **f** Resorbing, end-stage cataracts. **g** Anteriorly luxated cataract. **h** Inferiorly luxated cataract

Of particular interest were three kiwi found in our study that had bilateral and severe ocular lesions resulting in complete blindness. Surprisingly, these three individuals were found in good physical condition, assessed by complete physical examination and body condition scoring by a veterinarian with advanced training in avian medicine (JPM). The ocular lesions were also chronic in nature, suggesting that these birds had survived for months to years without vision. After our initial examinations, radio transmitter tracking revealed that the three blind individuals survived for at least 4 more years, and one of the birds had pair-bonded with a visual bird but it is unclear whether or not they successfully mated. There has only been one report of a blind, free-living bird: a kakapo (*Strigops habroptilus*) [8]. Additionally, a North Island brown kiwi (*Apteryx mantelli*) has been described to be blind, but was a captive bird [9]. Both cases, however, were limited to a single individual, and both the ophthalmic status and physical health were not reported in detail. Currently the health status of the endangered kakapo is closely monitored for conservation purposes, and no living kakapos are blind (personal examination JPM). The lack of reports of blind free-living birds in good health is a testament to the typical bird’s dependence on vision, and raises questions regarding the role of vision in kiwi.

Despite clear visual adaptations to maintain sight in a nocturnal activity pattern (e.g., rod-dominated retina) [5], the robust health status of the three blind kiwi described here suggests that vision is not necessary, at least not in the ecological niche these three kiwi occupied. So, how important are the eyes of kiwi, and what is their ultimate fate? Kiwi visual specializations may be remnants from a common ancestor that relied more heavily on vision for survival (e.g., moa), and thus we may be witnessing an example of adaptive regressive evolution [4, 5]. Unlike the kiwi, all vertebrate examples of known regressive evolution of the visual system inhabit areas devoid of light and have completely lost vision [4]. Kiwi could represent an intermediate stage of adaptive regressive evolution where the cost for maintaining a large eye is not well spent for what can be gained in low luminance on the forest floor [4]. Perhaps kiwi eye size and brain visual centers have adapted more readily than the retina and have thus diminished in their relative importance while leaving the retina relatively specialized [5]. This is plausible when one considers that decreasing eye size with a relatively constant decrease in the size of the pupillary aperture will result in similar light gathering ability. Additionally, complex central processing may not be necessary for crude detection of light level (and periodicity) provided the presence of sufficient intra-retinal processing. However, many of the ocular abnormalities described above are chronic in nature and are suggestive of this population being aged. The unique ecological niche of kiwi may contribute to the survivability of visually impaired, or even blind, individuals, and thus need not lead to adaptive change.

Resource competition among kiwi is low, which may contribute to the success of individuals with visual dysfunction. Visual ability does not seem to be related to foraging success by prey detection and capture given the good physical condition of the blind birds. Most birds that visually forage and can visualize their bill tip binocularly, but the long bills of kiwi are not visualized [1, 4]. This is supportive of the relative accentuation of other sensory systems and associated brain regions in kiwi: olfactory [7], auditory [6], and tactile [4, 7] sensory systems. Like some nocturnal mammalian species with olfactory specializations that forage on the forest floor [4, 10], kiwi may use vision to detect periodicity of day and night as a means of determining ideal activity time for foraging [5].

An alternative to adaptive evolution, for which direct evidence is currently lacking, to be considered is that perhaps the evolution of kiwi in the absence of natural mammalian predators has driven sensory allocation away from predator detection and towards sensory systems being more directed at nocturnal ground foraging and social interactions. Predator detection is an unrelenting challenge faced by most bird species, and is undoubtedly a major reason why profound ocular lesions in free-living birds are rare. The shaping of visual fields is multi-modal and is a balanced trade-off between foraging and anti-predatory behaviors [3]. For example, species that use non-visual cues while foraging tend to have wide visual fields (small blind areas, small binocular fields) for greater allocation toward the detection of predators [1]. In contrast, species that are heavily dependent on vision for foraging tend to have narrow visual fields (larger blind areas behind and above the head) but wider binocular areas [1]. The kiwi is the only known avian species that does not exemplify this pattern, having not only narrow binocular fields but also the narrowest visual fields (large blind areas) of any bird [4], presumably due to having evolved under little risk of predation. The eyes of kiwi have also not been reported to have significant degrees of movement, which is positively correlated with anti-predator behavior [3].

While it is apparent that kiwi are able to support themselves nutritionally in the complete absence of vision, we do not fully understand how kiwi utilize their visual system. Whether we are witnessing an intermediate stage of adaptive regressive evolution or a consequence of sensory drive due to a unique ecological niche is yet to be determined. Being a rare example of a flightless, nocturnal species of bird having evolved with no consistent natural predators, the kiwi represents an excellent opportunity to study the ecology and evolution of the visual system from a unique perspective.
